# Knowledge, perceived needs of continuous professional’s development, and associated factors among healthcare workers in East Ethiopia: a multi-health facility-based cross-sectional study

**DOI:** 10.1186/s12909-024-05486-z

**Published:** 2024-05-03

**Authors:** Tesfaye Assebe Yadeta, Ahmed Mohamed, Kerimo Behir, Addisu Alemu, Bikila Balis, Adera Debella, Shiferaw Letta

**Affiliations:** 1 School of Nursing and Midwifery, College of Health and Medical Sciences, Haramaya University, P.O. Box. 235, Harar, Ethiopia; 2 School of Medicine, College of Health and Medical Sciences, Haramaya University, Harar, Ethiopia; 3 School of Public Health, College of Health and Medical Sciences, Haramaya University, Harar, Ethiopia

**Keywords:** Continuous professional development, Awareness, Needs, Barriers, Healthcare workers, East Ethiopia

## Abstract

**Background:**

The Ethiopian Ministry of Health (EMOH) has recently introduced a Continuous Professional Development (CPD) program for healthcare workers to ensure they maintain the necessary competencies to meet the community’s health needs. However, there is limited information on healthcare workers’ knowledge and perceived need for CPD. This study aims to assess healthcare workers’ CPD knowledge, perceived needs, and factors associated with these in eastern Ethiopia.

**Methods:**

A health facility-based cross-sectional quantitative study was conducted from September 1, 2022, to October 30, 2022. Health facilities and study participants were selected using a simple random sampling technique. A total of 731 healthcare professionals were randomly selected. Data was collected using a self-administered questionnaire developed from national CPD guidelines. Data analysis was performed using the STATA statistical package version 14. A logistic regression model was used to assess the association between predictors and the outcome variable. Adjusted odds ratios with 95% confidence intervals were calculated to determine the strength of the association. A *p*-value < 0.05 was considered statistically significant.

**Results:**

In this study, 731 healthcare workers participated. Among them, 65.80% (95% CI: 62.35%, 69.24%) had knowledge of CPD, and 79.48% (CI95% 76.54, 82.41) expressed a strong perceived need for CPD. Female healthcare workers [AOR: 0.54 (95% CI: 0.37, 0.78)] and lack of internet access [AOR: 0.68 (95% CI: 0.47–0.97)] were predictors of knowledge of CPD. Age above 35 [AOR: 0.39 (95% CI: 0.17, 0.91)] and being female [AOR: 0.59 (95% CI: 0.40–0.87)] were predictors of a strong perceived need for CPD.

**Conclusion:**

The study found that there was a low level of knowledge about Continuing Professional Development among healthcare workers. The perceived needs of healthcare workers varied. It is important for health sectors and stakeholders to prioritize developing strategies that address knowledge gaps, particularly among female healthcare workers, improve access to the Internet for CPD resources, and address the diverse needs of professionals for effective CPD implementation.

## Background

Healthcare workers must continuously update their knowledge, skills, values, and attitude to meet the changing healthcare needs of their communities [1]. Continuing professional development encompasses all formal activities that healthcare workers undertake to maintain, update, and develop their competencies in response to public health service needs. In Ethiopia, CPD begins after the completion of basic or postgraduate health professional training, as outlined in the CPD guidelines [2, 3].

Healthcare workers’ knowledge and perceived need are crucial for the effective implementation of the CPD program [4]. The World Health Organization (WHO) recommends a need-based approach for CPD implementation, where health professionals and their organizations identify their CPD needs [5]. In Ethiopia, the Ministry of Health has recognized the importance of CPD in its policies and strategic plans for quality community health services since 2013 [6]. The ministry updated guidelines and implementation directives in 2018 to standardize, regulate, and accredit CPD mechanisms linked to health professionals’ licensure [3].

Despite efforts to standardize CPD accreditors and providers starting in 2020, there are only 219 CPD providers and 37 CPD accreditors established. The majority of these providers and accreditors are concentrated in the capital city, Addis Ababa, leading to uneven regional distribution [7]. This limited capacity of healthcare workers and slow progress in implementation since 2013 have negatively impacted the quality of healthcare delivery and patient outcomes.

In Ethiopia, it is mandatory for health professionals to obtain a professional practice license before practicing. This license must be renewed every three years after an evaluation of ethical and competence standards. Continuous professional development programs are essential for health professionals to stay updated on the latest advancements in their field and provide quality services. CPD guidelines aim to ensure that health professionals maintain their skills and knowledge to meet the expectations of delivering high-quality care [1, 3].

Continuous Professional Development intervention has been shown to enhance professional practice, improve patient outcomes [8–11], and elevate the quality of healthcare services for the community [12, 13]. However, the utilization of CPD in Ethiopia remains low [14]. Moreover, CPD is a relatively new concept in the Ethiopian healthcare system [3]. There is a lack of information regarding healthcare professionals’ knowledge and perceived need for CPD, as well as the factors influencing their CPD knowledge and perceived need in the eastern region of Ethiopia. Therefore, this study aimed to evaluate healthcare professionals’ CPD knowledge and perceived need, as well as the factors influencing these aspects in the eastern part of Ethiopia.

## Methods and materials

### Study area and period

The study took place in the East Hararghe Zone in Oromia Regional State, Eastern Ethiopia. The administrative center of this zone is Harar City, located 526 km from Addis Ababa, the capital city of Ethiopia. The zone is bordered by Bale to the southwest, the Hararghe Zone to the west, Dire Dawa Administration to the north, and the Somali Region to the north and east. It comprises twenty districts, three towns, eight public hospitals, and one hundred twenty-one health centers. The health professional human resource coverage is approximately 40.6% [7]. The study involved randomly selected health professionals working in health facilities in the East Hararghe Zone from September 1, 2022, to October 30, 2022.

### Study design and population

The study utilized a health facility-based quantitative cross-sectional study design. The study population consisted of healthcare professionals, including those in medicine, nursing, pharmacy, medical laboratory, midwifery, and public health, who were working in health facilities in Eastern Ethiopia. The study units were randomly selected healthcare workers.

### Sample size determination and sampling procedures

The sample size of 768 health professionals was determined using a single proportion population with an expected frequency of 50%, a confidence level of 95%, a confidence limit of 5%, and a design effect of two. A multistage sampling technique was employed, with 40 health centers and four hospitals selected randomly from a total of 121 health centers and eight hospitals in the East Hararghe Zone. Study participants were then randomly selected from the selected health facilities based on their proportionate load of health professionals [14].

### The data collection instruments and procedure

The data collection instruments were developed based on the National Continuing Professional Development Guidelines [3]. A structured self-administered questionnaire was pretested to ensure data quality. The pretest was conducted on 38 (5%) healthcare professionals in health facilities who were not part of the actual data collection. Questionnaires were revised based on the pretest findings. Data collection was carried out by ten Bachelor of Science health professionals, with supervision provided by five Masters of Science/Masters in Public Health holders. Both data collectors and supervisors underwent a 2-day intensive training session on the study’s objectives, procedures, data collection techniques, interviewing skills, and data collection methods. The principal investigator, co-investigators, and supervisors conducted thorough supervision, ensuring data completeness, accuracy, and consistency throughout the data collection period. Overall supervision was overseen by the principal investigators [14].

### Variables measurements

#### Knowledge of CPD

Knowledge of CPD referred as health care professionals have information of CPD. Healthcare professionals’ knowledge of CPD was assessed by asking the question, “Do you know about Continuous Professional Development?” Participants who answered “yes” were categorized as having knowledge (coded as 1), while those who answered “no” were categorized as not having knowledge (coded as 0).

#### Perceived CPD needs

Perceived CPD need is an individual’s assessment of the importance of continuing professional development. The question evaluates training needs in seven areas: clinical, management and leadership, communication, teaching/coaching, research, and ethics. Each area is scored from 0 to 4, with 0 indicating no need and 4 indicating the highest need. The total score is calculated by multiplying the number of boxes checked in each column by the corresponding value and adding the subtotals. The total score ranges from 0 to 28, with scores of 0–13 indicating a weak perceived need for CPD and scores of 14–28 indicating a strong perceived need [14].

#### Data analysis

The data was checked for completeness and consistency, then analyzed using STATA. Descriptive statistics were reported using tables and figures. A logistic regression model was used to assess the association between predictors and outcome variables. Multicollinearity was tested before entering variables into the model. Model fitness was assessed using the Hosmer–Lemeshow test. Crude Odds ratios were estimated for each variable in the bivariate analysis. Variables with *p*-value ≤ 0.2 were included in the multivariable analysis. Adjusted Odds ratios were calculated to assess the strength of the association, with a significance level of *p* < 0.05.

## Result

### Socio-demographic characteristics by CPD knowledge and perceived need

The data shows that a majority of healthcare professionals who knew about CPD were younger, with 64.63% under 25 years old and 67.59% aged 25–35 years. In terms of gender, 26.94% of males and 46.82% of females did not know about CPD. When it comes to the perceived need for CPD, 78.86% of those under 25 and 80.92% of those aged 25–35 had a strong perceived need. Among those with a strong perceived need, 82.97% were male and 73.41% were female. The study also found a significant association between knowledge of CPD and age (χ2 = 98.88, *p* < 0.001) and sex (χ2 = 29.75, *p* < 0.001) (Table 1).

**Table 1:** Socio-demographic Characteristics by CPD Knowledge and Perceived Need of Study Participants in East Hararghe, Eastern Ethiopia, 2022 (*n* = 731)

Variables	Knowledge of CPD *n* = 731			Perceived need of CPD *n* = 731		
	No *n*/%	Yes *n*/%	χ ^2^ (*P*-value)	Poor *n*/%	Good *n*/%	χ 2 (*p*-value)
age in year						
≤ 25	87(35.37)	159(64.63)	98.88(< 0.001)	52(21.14)	194(78.86)	3.36 (0.18)
26–35	141(32.41)	294(67.59)		83(19.08)	352 (80.92)	
≥ 36	22(44.00)	28(56.00)		15 (30.00)	35 (70.00)	
Sex						
Male	125(26.94)	339(73.06)	29.75 (< 0.001)	79(17.03)	385(82.97)	9.50 (0.002)
Female	125(46.82)	142(53.18)		71(26.59)	196(73.41)	
Marital status						
Married	162(34.47)	308(65.53)	0.04 (0.83)	90(19.15)	380(80.85)	0.15 (0.69)
Other	88(33.72)	173(66.28)		60(22.99)	201(77.01)	
Years of services						
≤ 5 years	122(35.78)	219(64.22)	1.69 (0.42)	75 (21.99)	266(78.01)	0.97(0.61)
6–10 years	85(31.25)	187(68.75)		51 (18.75)	221 (81.25)	
> 10 years	43(36.44)	75(63.56)		24(20.34)	94(79.66)	
Availability of internet						
Yes	127(30.02)	296(69.98)	7.78 (0.005)	79(18.68)	344(81.32)	2.09 (0.14)
No	123(39.94)	185(60.06)		71(23.05)	237(76.95)	
Distance from the main road						
≤ 20 km	45(36.89)	77(63.11)	0.46 (0.49)	22(18.03)	100 (81.97)	0.55 (0.45)
> 20 km	205(33.66)	404(66.34)		128(21.02)	481(78.98)	

Note: “χ 2” shows Chi square test along with 95% CIs

### Knowledge of CPD and activities that qualify for CPD

**Fig. 1:**
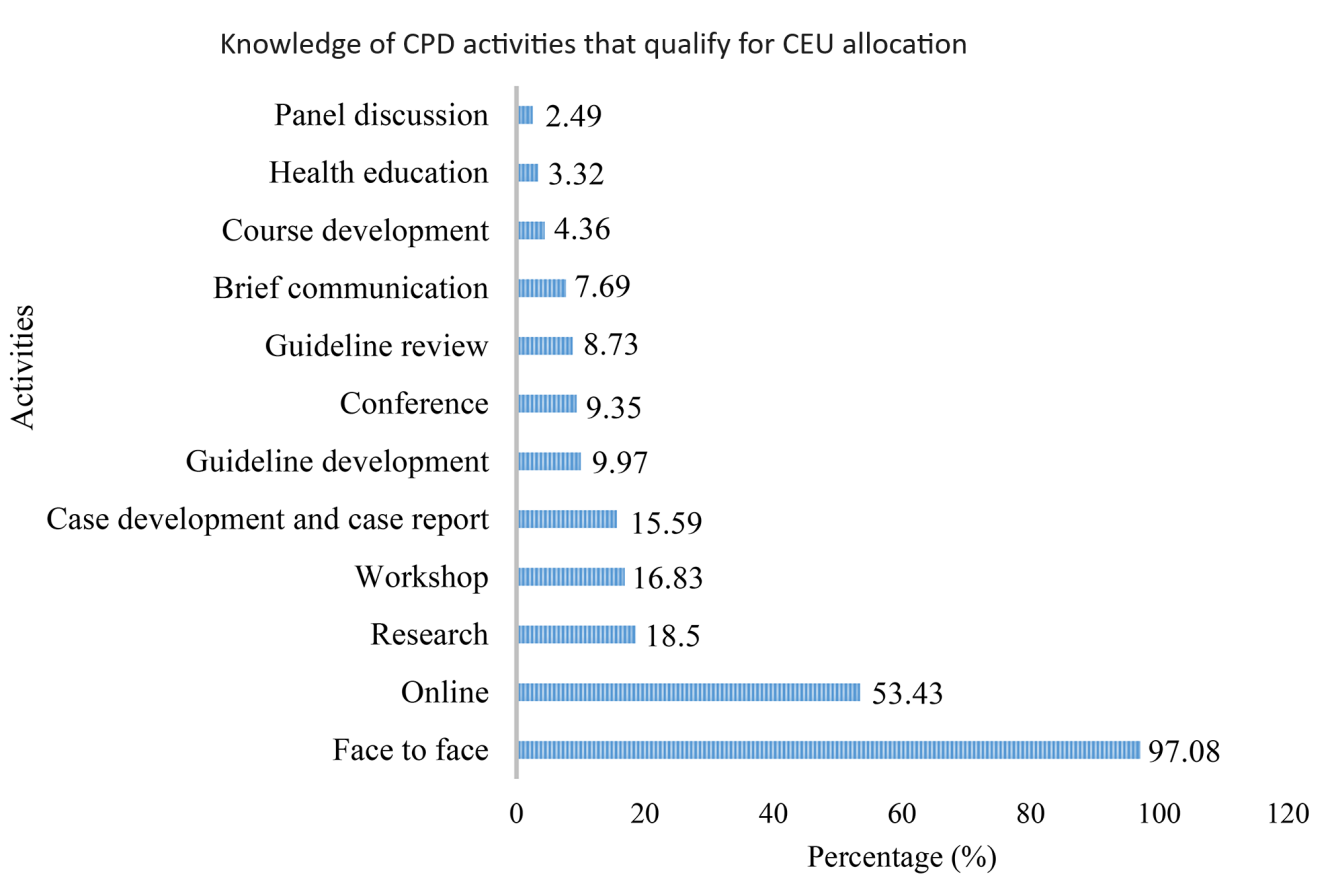
Knowledge of healthcare professionals regarding CPD activities that qualify for CEU allocation East Hararghe Zone, Eastern Ethiopia, 2022

The study assessed the knowledge of healthcare professionals regarding CPD activities that qualify for CEU allocation based on national guidelines. Out of 731 participants, 65.80% (95% CI: 62.35%, 69.24%) had CPD knowledge. Among the 481 participants who had knowledge of CPD, 97.08% recognized face-to-face training as a CPD activity, followed by online training (53.43%). Panel discussions were the least recognized CPD activity among the study participants (Fig. 1).

### Sources of information for CPD Knowledge

**Fig. 2:**
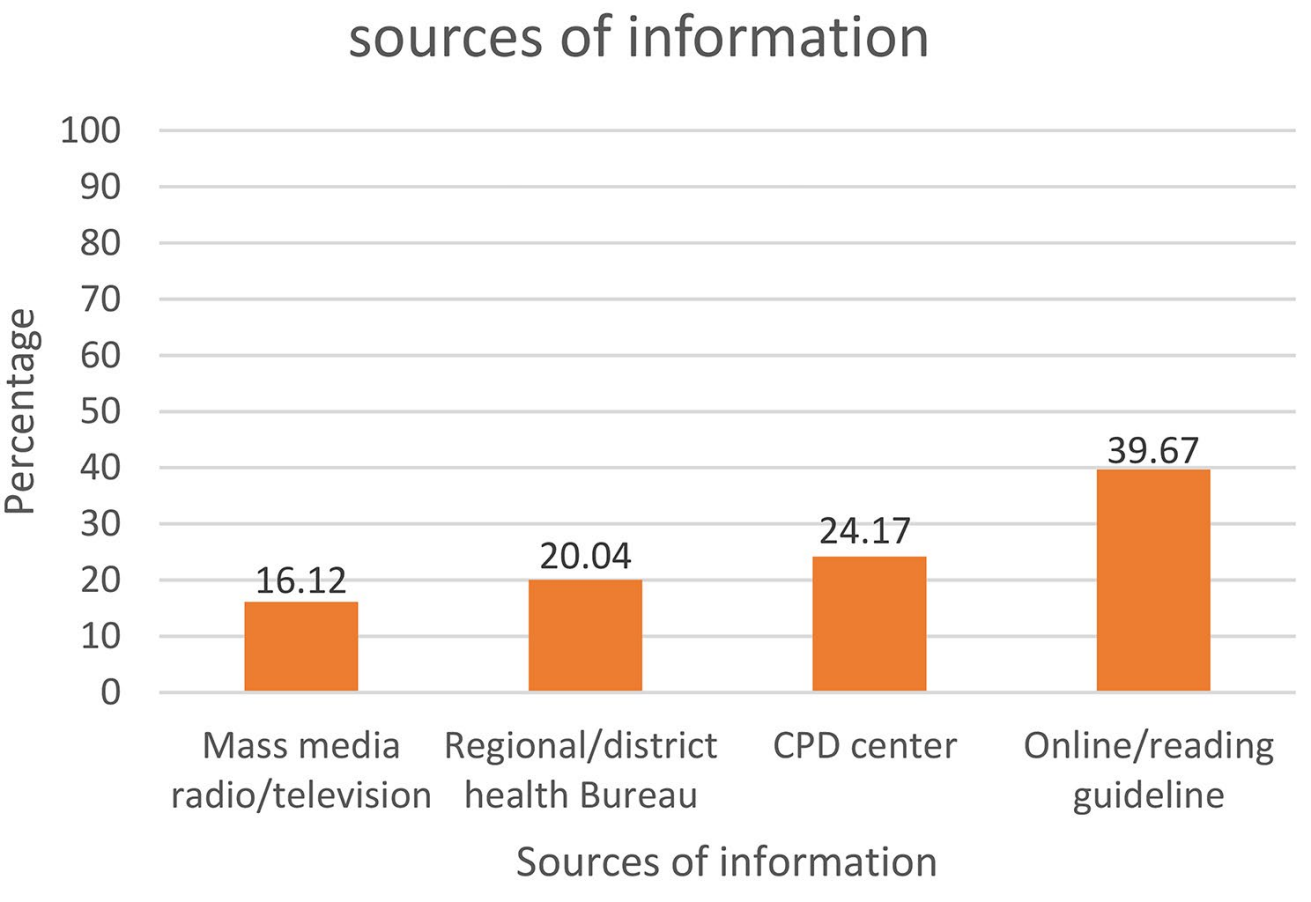
Sources of information for CPD Knowledge among healthcare workers East Hararghe Zone, Eastern Ethiopia, 2022

Participants who had CPD knowledge were asked about their sources of information. The majority of them reported getting information from online sources or reading guidelines (39.67%), followed by information from the CPD provider and accreditor center (24.17%) (Fig. 2).

### Perceived need for continuous professional development

Perceived CPD needs were assessed in seven areas of professional activities among 731 participants. The majority, 94.39%, expressed a need for training in clinical care, followed by 80.84% who identified a need for training in ethics and medico-legal issues. A composite analysis of the Likert scale responses indicated that 79.48% (95% CI: 76.54, 82.41) expressed a strong perceived need for CPD, while 20.52% expressed a weak perceived need for CPD (Table 2).

**Table 2:** Perceived need for continuous professional development (CPD) among study participants in East Hararghe, in 2022 (*n* = 731)

Variable	Need most	Need	Need Moderate	Neutral	Need no
	n(%)	n(%)	n(%)	n(%)	n(%)
Clinical care	645(88.23)	29(4.00)	16(2.19)	18 (2.46)	23(3.15)
Administration and leadership	337(46.10)	218(29.82)	36(4.92)	87(11.90)	63(8.62)
Patient communication skill	524(71.68)	22(3.01)	10 (1.37)	121(16.55)	54(7.38)
Teaching and coaching skill	321(43.91)	149(20.38)	58(7.93)	96(13.13)	107(14.63)
Research	333(45.55)	87(11.90)	128(17.51)	81(11.08)	102(13.95)
Ethics and medico-legal	557(76.19)	26(3.55)	8(1.09)	106(14.50)	34(4.65)
Data handling and interpretation	439(60.05)	9(1.23)	116(15.86)	117(16.00)	50(6.83)

### Predictors of knowledge of CPD

Female healthcare workers had a 46% lower knowledge of CPD compared to male healthcare workers [AOR: 0.54 (0.37, 0.78)]. Additionally, the lack of internet access was associated with a 32% lower knowledge of CPD compared to those with internet access [AOR: 0.68 (0.47–0.97)] (Table 3).

**Table 3:** Results of bivariate and multivariable logistic regression models on factors associated with Knowledge of CPD among healthcare workers in the East Hararghe Zone, Eastern Ethiopia, 2022

Variables	Knowledge of CPD *n* = 731		Crudes OR		Adjusted OR	
	No *n*/%	Yes *n*/%	COR	95% CI	AOR	95% CI
age in year						
≤ 25	87(35.37)	159(64.63)	1		1	
26–35	141(32.41)	294(67.59)	1.14	0.82, 1.58	0.88	0.57, 1.38
≥ 36	22(44.00)	28(56.00)	0.69	0.37,1.29	0.55	0.24, 1.26
Sex						
Male	125(26.94)	339(73.06)	1		1	
Female	125(46.82)	142(53.18)	0.41	0.30, 0.57	0.54	0.37, 0.78*
Marital status						
Married	162(34.47)	308(65.53)	1		1	
Other	88(33.72)	173(66.28)	1.03	0.75, 1.42	1.00	0.67, 1.50
Years of services						
≤ 5 years	122(35.78)	219(64.22)	1		1	
6–10 years	85(31.25)	187(68.75)	1.22	0.87, 1.71	1.22	0.78, 1.89
> 10 years	43(36.44)	75(63.56)	0.97	0.62, 1.50	1.23	0.67, 2.24
Availability of internet						
Yes	127(30.02)	296(69.980	1		1	
No	123(39.94)	185(60.06)	0.64	0.47, 0.87	0.68	0.47, 0.97*
Distance from the main road						
≤ 20 km	45(36.89)	77(63.11)	1		1	
> 20 km	205(33.66)	404(66.34)	1.15	0.76, 1.72	1.30	0.81, 2.0
Perceived need						
Perceived important	192(33.05)	389(66.95)	1		1	
Did not Perceived important	58(38.67)	92(61.33)	0.78	0.54, 1.13	1.09	0.71, 1.6

Note: “*” shows a statistically significant association of knowledge of CPD with independent variables along with 95% CIs, and a *p*-value < 0.05

### Predictors of the perceived need for CPD

Healthcare workers aged 36 or older were 0.39 times less likely to expresses a strong perceive need for CPD compared to those under 25 [AOR: 0.39 (0.17, 0.91)]. Additionally, female healthcare workers were 0.59 times less likely to express a strong perceive need for CPD compared to male healthcare workers [AOR: 0.59 (0.40–0.87)] (Table 4).

**Table 4:** Results of bivariate and multivariable logistic regression models on factors associated with the perceived need for CPD among healthcare workers in the East Hararghe Zone, Eastern Ethiopia, 2022

Variables	Perceived need of CPD *n* = 731		Crudes OR		Adjusted OR	
	Weak Perceived need *n*/%	Strong perceived need *n*/%	COR	95% CI	AOR	95% CI
age in year						
≤ 25	52(21.14)	194(78.86)	1		1	
26–35	83(19.08)	352 (80.92)	1.13	0.77, 1.67	0.82	0.51, 1.32
≥ 36	15 (30.00)	35 (70.00)	0.62	0.31, 1.23	0.39	0.17, 0.91*
Sex						
Male	79(17.03)	385(82.97)	1		1	
Female	71(26.59)	196(73.41)	0.56	0.39, 0.81	0.59	0.40, 0.87*
Marital status						
Married	90(19.15)	380(80.85)	1		1	
Other	60(22.99)	201(77.01)	0.79	0.54, 1.14	0.77	0.51, 1.16
Monthly income						
< 5000 Birr	36 (24.16)	113 (75.84)	1		1	
≥ 5000 Birr	114 (19.59)	468 (80.41)	1.30	0.85, 2.00	1.17	0.71, 1.92
Years of services						
≤ 5 years	75 (21.99)	266(78.01)	1		1	
6–10 years	51 (18.75)	221 (81.25)	1.22	0.82, 1.81	1.14	0.71, 1.84
> 10 years	24(20.34)	94(79.66)	1.10	0.65, 1.85	1.34	0.69, 2.59
Availability of internet						
Yes	79(18.68)	344(81.32)	1		1	
No	71(23.05)	237(76.95)	0.76	0.53, 1.09	0.81	0.56, 1.17
Distance from the main road						
≤ 20 km	22(18.03)	100 (81.97)	1		1	
> 20 km	128(21.02)	481(78.98)	0.82	0.50, 1.36	0.86	0.51, 1.44
Heard CEU						
Yes	46 (15.65)	248(84.35)	1		1	
No	104(23.80)	333(76.20)	0.59	0.40, 0.87	0.68	0.45, 1.01

Note: “*” shows a statistically significant association of perceived need of CPD with independent variables along with 95% CIs, and a *p*-value < 0.05

## Discussion

The study found that 65.80% of healthcare workers had CPD knowledge, while 79.48% perceived a good need for CPD. Female healthcare workers and access to the Internet were significantly associated with CPD knowledge. Additionally, healthcare workers above the age of 35 and female healthcare workers were significantly associated with a strong perceived need for CPD.

The Ethiopian Federal Ministry of Health requires all healthcare workers to renew their licenses by meeting the CPD national guideline requirement [1]. However, a study found that knowledge of CPD among participants was low at 65.80%, which is lower than reported in studies from Egypt (96.9%) [15], and Cameroon (98%) [16]. This discrepancy may be due to differences in the launch years of the CPD program and the availability of CPD services. In Ethiopia, updated guidelines and implementation directives were introduced in 2018 to standardize, regulate, and accredit CPD mechanisms for health professionals’ licensure [3]. Despite efforts to standardize CPD accreditors and providers starting in 2020, there are only 219 CPD providers and 37 CPD accreditors established. The majority of these providers and accreditors are concentrated in the capital city, Addis Ababa [7]. The slow progress in CPD implementation since its recognition in 2013 may be linked to the low CPD knowledge among health professionals [17]. Enhancing knowledge can improve healthcare workers’ practice [18]. Collaboration among the Ministry of Health, Regional Health Bureau, regulatory bodies, partners, CPD providers, and accreditors is essential to raise awareness. Knowledge-building initiatives such as workshops, conferences, multimedia use, and dissemination of national guidelines to the health sector require intensive efforts.

The study revealed that the perceived needs for CPD among healthcare workers vary. This finding aligns with similar studies conducted in Rwanda and Scotland. The importance of specific CPD activities is influenced by the healthcare worker’s job role and personal characteristics [19, 20]. In this study, the demand for clinical care CPD was notably high, which aligns with similar trends observed in other low-income countries [20, 21]. Healthcare workers’ positive perception of the importance of CPD activities had a beneficial impact on the intervention. It is crucial for the health sector to collaborate with healthcare workers to address their needs effectively. Conversely, if healthcare workers view the CPD program as irrelevant or unbalanced, it could diminish their enthusiasm for the program [21, 22]. Regulatory bodies should consider making CPD mandatory for relicensing to enhance the perceived need for CPD among healthcare workers and the health sector.

In this study, we observed that female healthcare workers had less knowledge of CPD compared to male health workers. This finding is consistence with the study done in Florida [23]. In low-income countries like Ethiopia, female healthcare workers often prioritize household activities, such as caring for their children and feeding their families [24]. This can hinder their participation in CPD activities due to challenges in accessing resources and digital skills [25]. To address this issue, a collaborative effort is needed from CPD providers, employers, government organizations, and civil society groups to create a supportive environment for women’s involvement in CPD. Improving readability by offering remote learning or flexible schedules, raising awareness about gender inequalities in CPD, and creating a supportive environment are crucial actions to take. Encouraging husbands to share more responsibilities in the family can also be beneficial [26]. .

In this study, we found that participants who did not have internet access had a lower understanding of CPD. This result is consistent with a study conducted in Canada [27], possibly because they lacked information about CPD [28]. The internet plays a crucial role in increasing CPD knowledge, as healthcare professionals frequently use online resources for information [20 28]. Accredited online courses are available, even within the country [17]. To enhance CPD knowledge, the government should enhance ICT infrastructure and internet access at health facilities. Moreover, it is essential to improve the digital skills of CPD providers and healthcare workers.

Changes in physical, learning, and performance abilities can impact the perceived need of CPD in older individuals [29]. In our study, we observed that older health professionals had a lower perceived need for CPD compared to younger counterparts. When designing CPD programs, it is important to address the motivation of older adults in the healthcare field to enhance their engagement, as motivation is a key factor in learning [30]. Factors such as physical limitations, information overload, and difficulties in customizing user interfaces can create barriers to older adults’ participation in CPD programs [31].

Understanding the awareness, perceived needs, and barriers to CPD among health professionals is crucial for developing effective CPD programs that meet their professional needs and help them improve their practice [19]. This study provides valuable evidence for decision-making in the healthcare sector, which is often lacking in many countries [21]. The global healthcare system is constantly evolving with advancements in technology, diagnostic tools, treatment methods, and health promotion strategies. Health professionals must engage in CPD to meet the changing needs of the community. Policy makers and stakeholders should prioritize CPD implementation and adherence to national guidelines. Female health workers, particularly in low-income countries like Ethiopia, face challenges balancing work and home responsibilities, impacting their access to CPD. However, female health professionals play a crucial role in providing quality patient care and should be supported in their professional development. The authors suggest that researchers assess the impact of educational interventions on improving knowledge and engagement in CPD among healthcare workers at health institutions using a theoretical model.

### Strength and limitation

The study included multiple health facilities and professionals, following national CPD guidelines to develop the tool. The results can be generalized to healthcare workers in the study area. However, collecting data on dependent and independent variables simultaneously makes it challenging to establish causal relationships. Another limitation is the reliance on self-reported data, which may be subject to recall bias, social desirability bias, and interviewer bias. To mitigate these biases, data collection procedures were carefully implemented, including training data collectors and close supervision.

#### Conclusion

The study found that there was a low level of knowledge about Continuing Professional Development (CPD) among healthcare workers. The perceived needs of healthcare workers varied. It is important for health sectors and stakeholders to prioritize developing strategies to increase awareness about CPD, particularly among female healthcare workers, improve access to the internet for CPD resources, and address the diverse needs of professionals for effective CPD implementation.

## Data Availability

Data availability statement: Upon a reasonable request the data will be obtained from the corresponding authors.

## Abbreviations

AOR: Adjusted Odds Ratio
CEU: Continuous educational Unit
CI: Confidence intervals
COR: Crud odds ratio
CPD: Continuous Professional Development
FMOH: Federal Minster of Health
WHO: World Health Organization
